# Cod Liver Oil

**Published:** 1890-05

**Authors:** 


### COD LIVER OIL.

Cod liver oil is, as its name indicates, obtained from the livers of codfish. It is an agent which could hardly be dispensed with, being a nourishing tonic of exceeding value. Many people have an idea that consumption is the one disease for which it is peculiarly adapted, and they fail to recognize the fact that it is equally efficient in many other affections. Hence, when physicians prescribe it, patients at once assume that they have trouble with their lungs, says the Boston *Herald*.

The accepted list of diseases in which cod liver oil is of special efficacy is much larger than it was a score of years ago. Undoubtedly, physicians in old times, in attempting to combat disease, often used drugs which depressed and reduced the vital powers, doing thereby more harm than good. All that is changed now. Physicians of the present may be said to ignore to a certain extent, the disease, but nourish and keep up—" restore the life that is being drained, build up the tissues being wasted." Cod liver oil is practically a food, and as such only, does it act. It nourishes and fattens wasted and wasting bodies, and in that way it often checks the progress even of pulmonary consumption.

Among the many affections in which it is given, is nervous debility. In some coughs, too, even where the lungs are perfectly sound, it proves admirable, and often cures the same. Its taste is so disagreeable that comparatively few patients can take it, a fact much to be deplored. Many are the ways devised to make it less unpleasant, flavoring it with peppermint, mixing it with coffee, rinsing the mouth first with brandy or whiskey, pouring it into the froth of the beer. Some recommend that it be salted and peppered and then " bolted down," afterward the mouth to be rinsed with tincture of myrrh and water. Lately, it has been suggested that a few grains of salt be dropped on the tongue before taking cod liver oil, as by that means it will be rendered palatable. Or a bite of pickle before and after taking the oil, will render it more acceptable.

